# Larval Dispersal Modeling of Pearl Oyster *Pinctada margaritifera* following Realistic Environmental and Biological Forcing in Ahe Atoll Lagoon

**DOI:** 10.1371/journal.pone.0095050

**Published:** 2014-04-16

**Authors:** Yoann Thomas, Franck Dumas, Serge Andréfouët

**Affiliations:** 1 Institut de Recherche pour le Développement, unité de recherche CoRéUs, Nouméa, New Caledonia; 2 Institut Français de Recherche pour l’Exploitation de la Mer, unité DYNECO, Plouzané, France; CSIR- National institute of oceanography, India

## Abstract

Studying the larval dispersal of bottom-dwelling species is necessary to understand their population dynamics and optimize their management. The black-lip pearl oyster (*Pinctada margaritifera*) is cultured extensively to produce black pearls, especially in French Polynesia's atoll lagoons. This aquaculture relies on spat collection, a process that can be optimized by understanding which factors influence larval dispersal. Here, we investigate the sensitivity of *P. margaritifera* larval dispersal kernel to both physical and biological factors in the lagoon of Ahe atoll. Specifically, using a validated 3D larval dispersal model, the variability of lagoon-scale connectivity is investigated against wind forcing, depth and location of larval release, destination location, vertical swimming behavior and pelagic larval duration (PLD) factors. The potential connectivity was spatially weighted according to both the natural and cultivated broodstock densities to provide a realistic view of connectivity. We found that the mean pattern of potential connectivity was driven by the southwest and northeast main barotropic circulation structures, with high retention levels in both. Destination locations, spawning sites and PLD were the main drivers of potential connectivity, explaining respectively 26%, 59% and 5% of the variance. Differences between potential and realistic connectivity showed the significant contribution of the pearl oyster broodstock location to its own dynamics. Realistic connectivity showed larger larval supply in the western destination locations, which are preferentially used by farmers for spat collection. In addition, larval supply in the same sectors was enhanced during summer wind conditions. These results provide new cues to understanding the dynamics of bottom-dwelling populations in atoll lagoons, and show how to take advantage of numerical models for pearl oyster management.

## Introduction

Understanding the population connectivity of marine species is necessary to fully comprehend population dynamics, community dynamics and structure, genetic diversity, and the resilience of natural populations to human exploitation [1]. Population connectivity is driven by interactions between organism physiology, morphology and behavior, and the biological and physical environments [2]–[4]. The main factors explaining spatial and temporal variability in recruitment are: (1) the larval supply, dependent on broodstock density, fecundity and spawning seasonality [5], (2) the larval dispersal, driven by the currents and organism behavior [6], (3) the larval development, primarily controlled by temperature and trophic resources availability [7], (4) the larval mortality, as a consequence of predation, physiological stress (e.g., temperatures, salinity or oxygen stress), disease and parasites [8], (5) the habitat suitability [9] and finally (6) post-settlement survival, which depends on delay of metamorphosis, biological and physical disturbances, hydrodynamics, physiological stress, predation, and competition [10]. Given the complexity of the processes controlling recruitment, and given the inherent difficulties in measuring larval dispersal and development in the field, biophysical models are increasingly used to explore how biological and physical factors influence larval dispersal and settlement [2], [3], [11]–[13]. Connectivity modeling studies typically aim to identify sources (i.e., where larvae are spawned), destinations (i.e., where larvae settle), and fluxes. If *in situ* recruitment data are available, a sensitivity analysis can identify the most important factors explaining the observed variability [14], [15]. However, very few studies have used good validation and forcing data.

The main objective of the present study was to identify the factors most affecting the larval dispersal kernel (defined as the probability function of the dispersal distance) of the black-lip pearl oyster (*Pinctada margaritifera* (L.) *var. cumingii* (J.)). The study is conducted at the scale of a specific atoll lagoon (Ahe atoll), in the context of *P. margaritifera* culture, but it also contributes to the general understanding of the dynamics of bottom-dwelling populations in atoll lagoons. The black-lip pearl oyster is found throughout the Indo-Pacific region, from the Red Sea to Central America. This species is particularly abundant in Polynesian archipelagoes and is the foundation species of the black pearl industry, one of the main economical resources of French Polynesia and Cook Islands since the 1990s. Intensive research has taken place in Polynesian lagoons in the past decades, enhancing knowledge on atoll lagoon hydrobiology, lagoon planktonic food resources, and *P. margaritifera* ecophysiology (see review in [16]). Recently, larval ecology was studied in priority to better understand spat collecting variability [7], [17]. Indeed, the black pearl industry production requires steady rates of spat collection. This process yields the necessary oysters that will be subsequently grafted to provide the valued black pearls. The most recent findings on *P. margaritifera* larval ecology have been acquired in Ahe atoll in the Tuamotu Archipelago, French Polynesia [16].

To model larval dispersal, the first step was to implement, calibrate and validate for the Ahe lagoon, a 3D Eulerian transport model coupled with an empirical vertical swimming sub-model [18], [19]. This biophysical model was validated against a series of larval census campaigns [19]. These first studies showed the good agreement between observed and simulated dispersal kernels. They also showed the significant influence of the wind on larval dispersal, thus a major driver of the potential connectivity of *P. margaritifera* in Ahe lagoon. The present study aims to refine these results by looking at how various forcing variables affect the pearl oyster larvae dispersal kernels both spatially and temporally. For this, the influence of climatological wind forcing, depth and location of larval release, destination location, vertical swimming behavior, pelagic larval duration factors and broodstock location was investigated, to draw a realistic picture of Ahe atoll lagoon-scale connectivity. The consequences of our results for pearl oyster culture management are also discussed.

## Materials and Methods

### Ethics statement

This work did not involve the manipulation of animals, thus it did not involve endangered or protected species. Modeling methods did not require specific permission from any relevant body as they are harmless and meet all applicable standards for the ethics of experimentation and research integrity.

### *Pinctada margaritifera*: ecology, aquaculture rearing stock and natural stock

We present hereafter, useful background information on *P. margaritifera* biology, ecology and culture for the connectivity modeling parameterization.

*P. margaritifera* is a protandrous hermaphrodite, male in early life and female later on, with sex ratio being balanced by age. *P. margaritifera* maturity is reached during the first year, followed by an important gonad development during the second year [20]–[22]. Gametogenesis is quick (1 month), and is observed throughout the year with a significant asynchronism. However, austral summer is the more favorable period [22]. At each spawning event, females can propagate up to 40–50 million eggs (50 µm) and males 10–100 time more spermatozoa (5 µm). Fecundation occurs in the water. The first larval stage (D-larva, 80 µm), is reached after 24 h. A ciliate organ (the velum) allows swimming and feeding activities. The pelagic larval duration (PLD) may vary from 15 to more than 30 days [7]. After metamorphosis, young spats fix themselves to the substratum with their byssus, between 0 and 50 m depth. Adult life duration may be more than 12 years, with a theoretical maximum length of 18 cm, but larger shells are commonly seen on several atolls [23], [24]. Preferred substrates are along the flanks of coral pinnacles and on deep coral patches, as well as debris from coral and mollusk shells on lagoon sandy bottom. Oysters were naturally abundant in deep atoll lagoons such as Manihi, Scilly, Takapoto atolls and Mangareva island [25], [26]. Historical stock assessments revealed a population peak around 20–40 m depth. In Takapoto atoll, the bulk of the natural stock, estimated at 4.3 million oysters in 1995, was found around 30 m depth [27].

In French Polynesia, pearl culture spread across 27 islands, among which 15 developed a spat collecting activity. Spat collection supports the entire Polynesian production. Indeed, the pearl culture relies entirely on the supply of wild juveniles collected on artificial substrates. Typically, collectors are placed along 200 m longlines submerged at 5 to 10 m depth. Spat collection devices can be set anywhere depending on the farmer's experience (or estimate). After 12-to 24 months, the oysters on the collectors have grown by 5–10 cm and are afterwards set on oyster-rearing chains suspended on lines at 6 to 10 m depth. These 200 m oyster-rearing chains support between 4 000 to 10 000 oysters. After grafting 18 month-old oysters, the last stage is the pearl harvest. This occurs after another rearing period of about 18 months. Unlike spat collection areas, rearing sectors are legally defined by marine concessions boundaries, which are periodically controlled and mapped. Rearing concession capacities are legally limited to a maximum of 12.000 oysters per hectare. Therefore, the rearing stock and its location can be estimated precisely for any given atoll.

### Study site: Ahe atoll and lagoon

**Figure 1:**
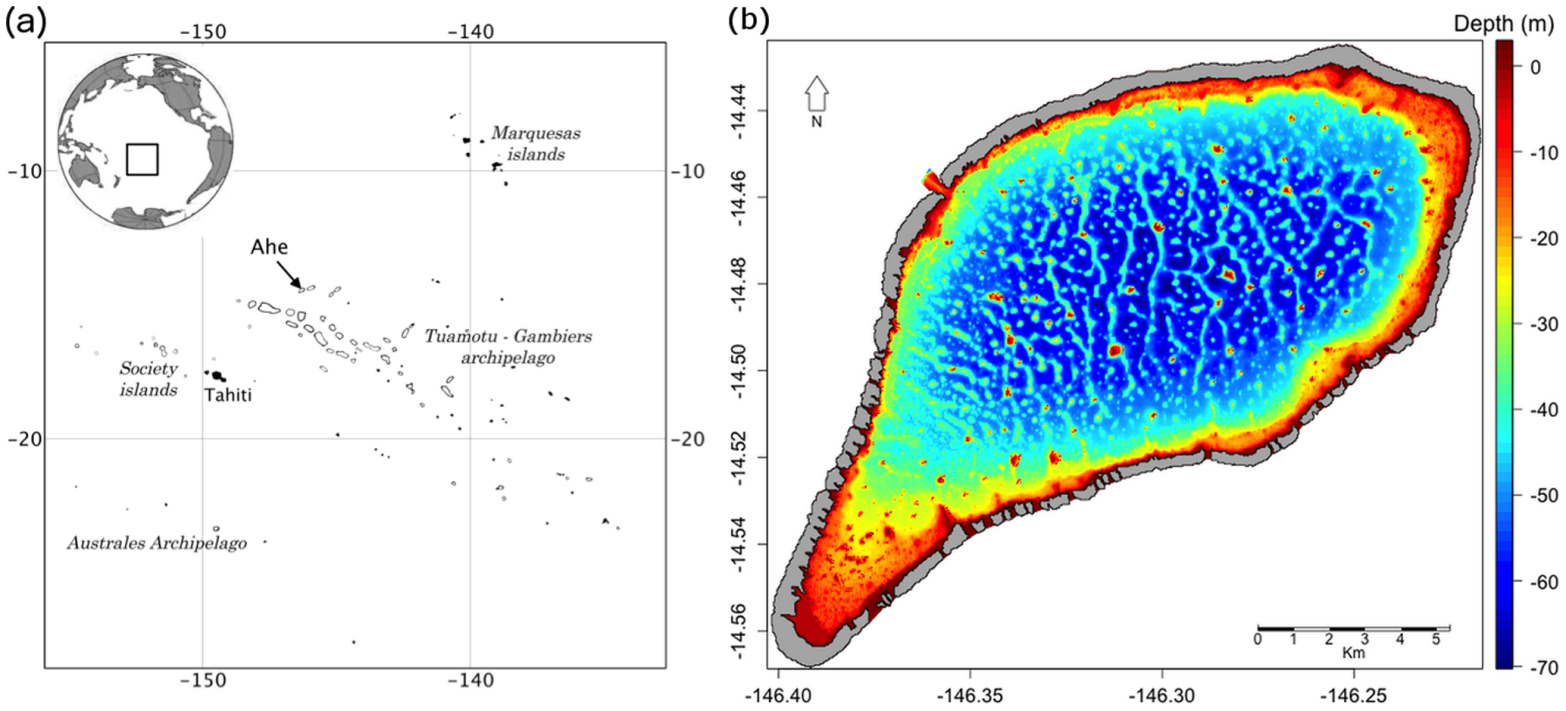
The Ahe atoll. (a) Ahe atoll location and (b) morphology and bathymetry of the Ahe lagoon.

Ahe atoll is located in the northwestern part of the Tuamotu Archipelago (14.48S–146.30W), about 500 km northeast of Tahiti (Figure 1a). The lagoon is a 142-km^2^ deep-water body with an average depth of 41 m, reaching up to 70 m depth. Numerous pinnacles rise to the surface. The deeper areas are made of honeycomb-like cellular structures. The volume of the inner water body is 5.9×10^9^ m^3^.

The lagoon is an almost closed water body connected to the open ocean through a 11-meter deep, 200-meter wide, pass located on the north-west side of the atoll rim (Figure 1b). Several reef-flat spillways connect the ocean to the lagoon. These spillways, named hoa (a Tahitian designation now part of the formal geomorphology vocabulary), are about 30 cm deep, between 10 and 300-meter wide, with a total cumulated width of 4 km. Thus, they represent about 5% of the rim perimeter (77 km). Hoa are only present on the southern side and on the northwestern side of the rim. Other rim sections are completely closed to water exchanges. All year round, Ahe receives wind waves generated locally by dominant easterly trade winds, which are typically stronger from April to October [28]. In contrast, Ahe is subjected to an attenuated swell regime due to its northward position, in the lee of the large Tuamotu atolls that block the predominant south swells all year round [28].

Ahe atoll's lagoon water circulation is driven by wind [18]. The primary circulation pattern is a downwind surface flow and a returning upwind deep flow. Dumas et al. (2012) [18] described two main barotropic circulation structures under climatological tradewinds. First, a north large anticlockwise circulation pattern occupied two-thirds of the water body with residual currents of around 5 cm.s**^−^** ^1^. Second, a weaker clockwise circulation pattern occurred in the south. The renewal time of the water body has been estimated at 252 days. The average e-flushing time, which corresponds to the time needed to decrease a concentration of tracers (e.g. larvae) by a factor e = 2.718, was 80 days. It ranged from 50 days in the vicinity of the pass to 140 in the center of the northern circulation cell [18].

### Larval production of Ahe lagoon

**Figure 2:**
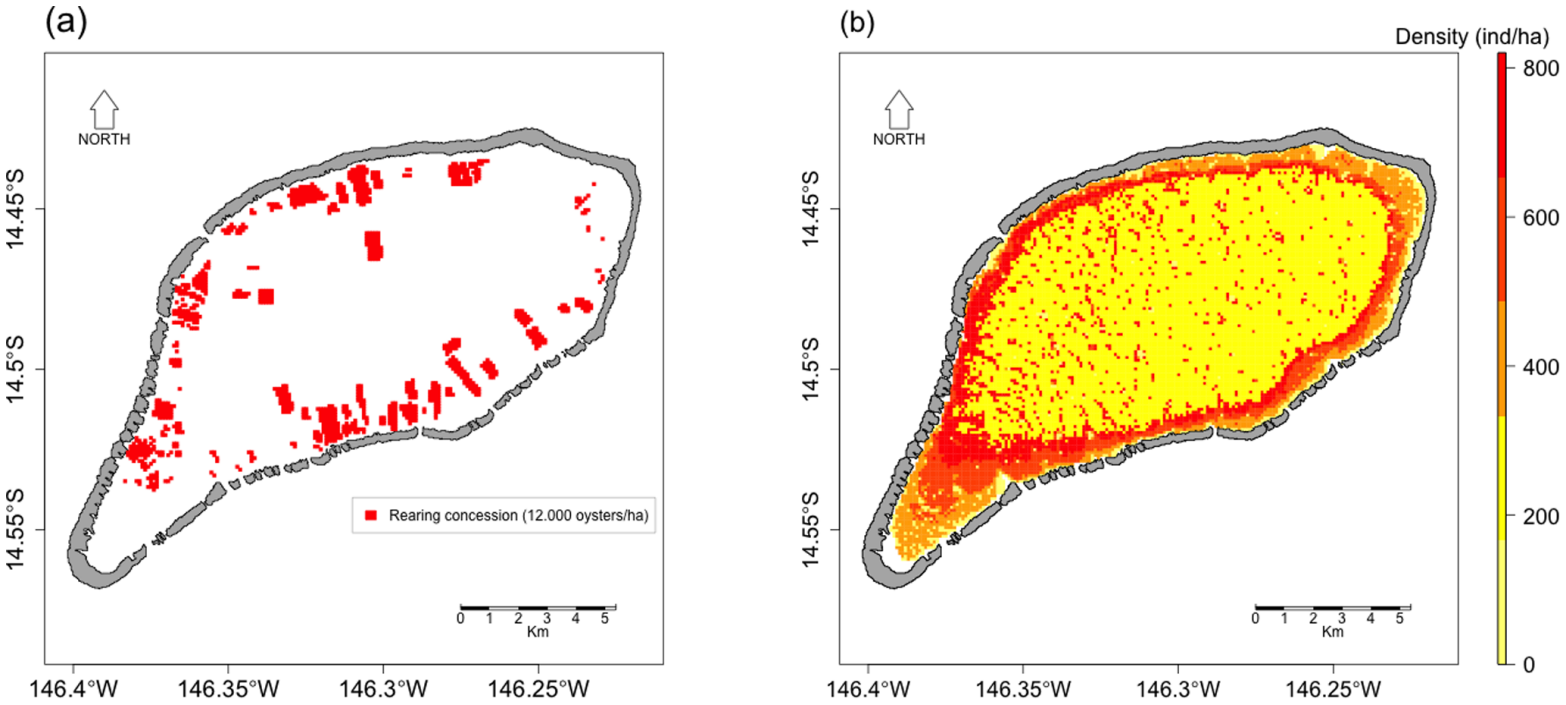
The pearl oyster broodstock. Maps of (a) the pearl oyster rearing concessions, (b) the natural pearl oyster density. The pearl oyster concessions correspond to the situation in 2012, with 12.000 oysters/ha (source: Direction des Ressources Marines). The natural pearl oyster density is an estimation after [27] (scale: oysters per hectare).

Larval production (*W_s_*) was assumed to be directly linked to the broodstock. We did not consider a potential spatial heterogeneity of fecundity. Two stocks were considered: natural and reared. The cultivated stock was mapped using the official concession boundary information (Figure 2a, Marine Resource Direction, pers. comm.) and considering 12.000 oysters per hectare of concession. Conversely, the distribution of the natural stock was still unknown in Ahe lagoon at the time of this study. Therefore, we applied the density by depth level found by Zanini and Salvat (2000) [27] in Takapoto atoll (Table 1, Figure 2b). Table 2 provides the reared and natural stocks for each of the 12 spawning sectors considered here (defined below).

**Table 1:** Density of wild pearl oyster according to the bathymetric level (after [27]).

Bathymetric level	Density (oyster/100 m^2^)
0–10 m	1
10–20 m	3.6
20–30 m	5.2
30–40 m	8.2
>40 m	2.5

**Table 2:** Estimation of the reared and natural oyster stock, cumulated by spawning site (see Figure 3).

Spawning site	Reared stock	Natural stock
1	1.092.000	379.280
2	972.000	633.580
3	2.364.000	450.540
4	1.848.000	518.370
5	1.212.000	452.690
6	0	347.360
7	2.616.000	449.960
8	0	297.540
9	2.184.000	413.620
10	864.000	381.790
11	948.000	431.570
12	264.000	399.560
*Total*	*14.364.000*	*5.155.860*

Two different release scenarios were further considered. First, the reared population emitted larvae between 5 and 10 m depth, where longlines are typically immerged. Second, the natural stock emitted larvae from the bottom layer, where the natural stock is located.

### Spawning and destination sites

**Figure 3:**
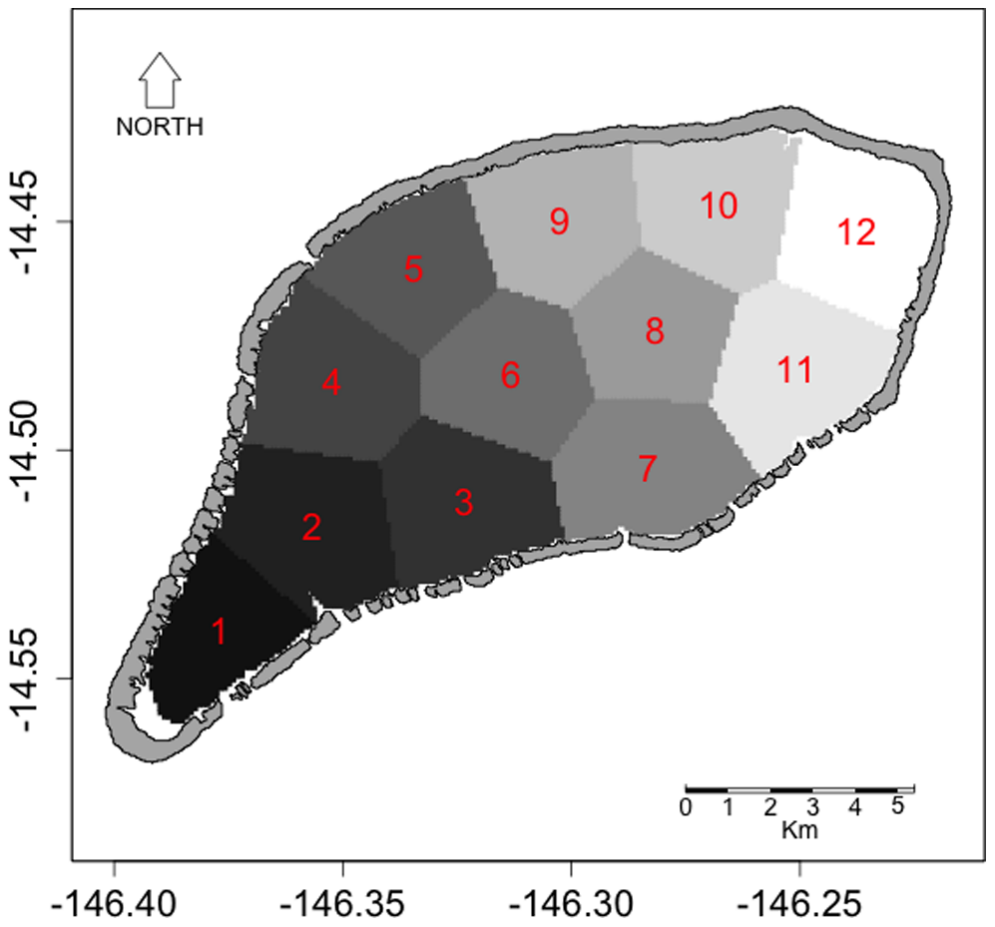
Map of the 12 spawning sites and destination locations, of the connectivity study. The extent of each sector was obtained through a clustering method performed on the longitudes and latitudes of the model grid. Only the near shore sectors, with less than 5

Twelve sectors of similar surface areas, representing both spawning and destination sites, were defined for the connectivity analysis (Figure 3). The extent of each sector was obtained by clustering the longitudes and latitudes from the model grid. The number of sites was a compromise; a high enough number chosen to realistically represent the various parts of the lagoon, described in previous studies [19] and also low enough to optimize the model computing time. Only the near shore sectors, with less than 5 m depth and thus unsuitable for farming activity, were not included. These shallow sectors and the open ocean were nonetheless considered in the connectivity study as destination locations only. Shallow sectors and open ocean were used to estimate the loss rate and export rate respectively.

### The pelagic larval duration (PLD)

There is currently very little *in situ* information on PLD for *P. margaritifera*. Most of our knowledge comes from hatchery experiments. They provided a mean PLD of 21 days [29]. However, depending on environmental conditions (e.g., temperature, food concentration), PLD may vary from 15 to more than 30 days [7]. Connectivity patterns were thus computed after 15, 20, 25 and 30 days of simulation. This indirectly provided the potential effects of food and/or temperature on the larvae and *in fine* on connectivity.

### Bio-physical transport model

Larval transport and dispersal were simulated with the 3-D hydrodynamic model MARS3D, which resolves the ocean dynamics equations [30]. A full description of the bio-physical larval transport model can be found in Thomas et al. (2012) [19] and model implementation and validation are explained by Dumas et al. (2012) [18]. In short, the model is constrained by a horizontal cell size of 100 m by 100 m. The vertical resolution of the model includes 23 sigma-vertical layers. These sigma-vertical layers are tightened close to the bottom and to the surface in order to better represent velocity gradients at the interface layers.

The hydrodynamic model was coupled with an advection/dispersal module, itself integrating a model reproducing the larvae dial vertical swimming behavior. The larval transport followed an Eulerian scheme since the state variables were calculated at fixed locations. Larval distribution was thus described as a grid of larval concentrations, transported by the water flow network and by the vertical swimming displacement. The larvae exported to sectors unsuitable for farming activity, into the ocean, the pass, and shallow waters (less than 5 m-deep), can be remobilized into the global larval pool via inbound exchanges through the pass and the hoa, or from the shallow waters to the deeper one.

The swimming model simulated the vertical displacement velocity following a sinusoid centered on 0, with a positive velocity (going up) during the night and a negative velocity (going down) during the day [19].

Biophysical models are able to simulate dispersal accurately, but they require assumptions on relevant physical processes, boundary conditions, atmospheric forcing, and larval behavior [2]. The present biophysical model was validated against numerous field records [18], and good agreement between observations and simulations of larval concentrations was achieved, both at the vertical and lagoon scales [19].

### Potential and realistic connectivity

Following Watson et al., (2010) [13], the potential connectivity is defined as the probability of larval transport from a spawning site (*i*) to a destination location (*j*). Conversely, realistic connectivity is defined as the actual (or modeled) number of larvae that travel from *i* to *j*. We intentionally modified the term “realized” used by Watson et al. (2010) [13], by “realistic”, considering that the realized connectivity depends on effective recruitment, after settlement and post-settlement [4], [31]. In contrast, the realistic connectivity only depends on dispersal processes until the stage of competent larvae. The reason to compare the potential and the realistic connectivity is to assess the relative importance of the spatial distribution of the broodstock (i.e., natural and reared) on the patterns of larval connectivity. In other words, the potential connectivity was calculated following a homogeneous distribution of spawners, while realistic connectivity accounts for the spatial distribution of the stocks.

The potential connectivity *P_sd_* was calculated according to the physical (*i.e.,* hydrodynamics) and behavioral (*i.e.,* vertical migration) factors, following: 
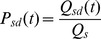



Where *Q_sd_* is the number of larvae found in the destination location *d* at time *t* and coming from the spawning site *s*, and *Q_s_* is the total number of larvae released in the spawning site *s*. For the simulations, the initial larvae concentration in the spawning sites was set at 100 individuals per hectare.

In a second step, the realistic connectivity *L_sd_* at time *t* was defined as the product of potential connectivity *P_sd_*, and larval production *W_s_*, which corresponds to the number of spawners in the considered spawning site:
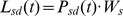



Fecundity and mortality rates were not explicitly included in this study. The realistic connectivity thus gives a theoretic number of larvae, only dependent on the number of adults in the different spawning sites.

### Weather regimes

To evaluate the effects of wind on larval dispersal and lagoon-scale connectivity, we identified a number of typical 30-day realistic wind sequences for the simulations using a long-term meteorological reanalysis. Meteorological dataset from ERA-Interim analysis (http://www.ecmwf.int/research/era/do/get/era-interim) were extracted daily at the closest grid point to Ahe from 1st January 1979 to 31th December 2011. The 30-day sequences were selected with an overlap of 20 days between each, giving 1203 sequences for the entire 32-year period.

Principal component analysis (PCA) and clustering techniques are widely used to identify climatological regimes in observations and model results [32]. PCA was performed on the wind stress components since it is linearly related to the transport within Ekman layer. It could be expressed as:
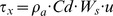


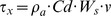



where *ρ_a_* is the air density, *Cd* the drag coefficient, *W_s_* the wind speed and (*u*, *v*) its zonal and meridian components. To a certain extent (i.e., under moderate wind conditions and in a medium where sea roughness remains moderate, like in an enclosed area such as a lagoon), *Cd* can be considered constant (e.g., 0.0016 following Deacon and Webb 1962 [33]). Under this hypothesis, our classification could be performed using:
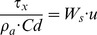


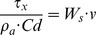



which scale homothetically with the wind stress itself and thus does not influence the classification. *W_s_*, *u*, and *v* come directly from the ERA interim re-analysis data set.

In order to optimize the PCA calculation and still preserve the intra-sequence variability (e.g., distinguish a permanent null sequence from “loop shaped” sequence), each sequence was summarized into 15 sub-sequences of 2-day averages. This was found to be the best trade off. PCA was performed on non-standardized dataset, in order to maintain the absolute variability.

A k-means clustering was performed on the PCA scores using the *pam* function of the *cluster* package in R software [34]. No significant gain in cluster dissimilarity was found beyond 12 clusters, and 12 was kept as the optimal number of clusters, corresponding to a 10% threshold in dissimilarity. The real wind sequence the closest to the barycenter of each cluster was selected as reference for the cluster. This finally yielded the 12 wind regimes used for the different connectivity scenarios.

Finally, the occurrence probability was calculated for each cluster as the ratio between the number of sequences included in each cluster and the total number of sequences (i.e., 1203).

### Connectivity scenarios, statistical analysis and connectivity matrices

Table 3 summarizes the range of biophysical factors used in the different connectivity scenarios and the number of treatment per factor.

**Table 3:** Summary of the factors tested in the connectivity analysis.

Factor	Treatments	Nb. of treatments
Larval release	5–10 m depth/bottom	2
Swimming	With/without	2
Wind regime	12 sequences of 30 days	12
Spawning sites	12 spawning sites	12
Destination location	12 destination locations + intra-lagoon shallow water + open ocean	14
PLD	d15, d20, d25, d30	4

To test the effect of these factors on connectivity results, we applied ANOVA using *aov* function of the R software [35]. Since ANOVA requires normal data, the connectivity output data were transformed. The potential connectivity probabilities (*P_sd_*) were normalized following a Arcsine(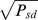) transformation. The realistic connectivity results (*L_sd_*) were subjected to a BoxCox transformation: 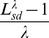 with lambda estimated at 0.3.

A transition probability matrix formalized the potential connectivity. For the realistic connectivity, the theoretic number of larvae replaced the probability in the transition matrix. To rank the different sites in terms of ‘source’ and ‘sink’ potential, the cumulated connectivity of each spawning site (i.e., column of the matrices) and destination location (i.e., row of the matrices) are computed for both the potential and realistic connectivity matrices. To represent the spatial heterogeneity of cumulated connectivity, the summed data were standardized using the standard score z calculation: z = (V–MV)/SD, where V, MV and SD are respectively the data value, the mean value and the standard deviation.

## Results

### Wind regimes

**Figure 4:**
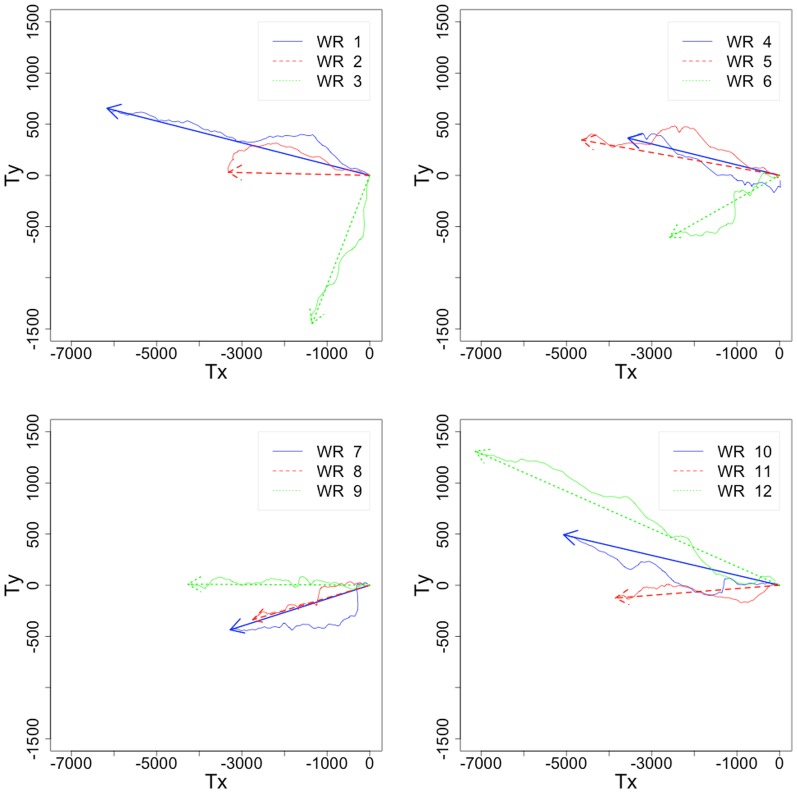
Hodographs of the 12 wind regimes. These graphs are performed with the wind stress components Tx and Ty. Arrows give the mean pattern for the 30 days. The numbers correspond to the wind regime indices (see Table 4).

Twelve wind regimes (WR) of 30 days each were identified with the clustering (Table 4). The overall frequencies of each WR are between 4.9% (WR 12) and 13.3% (WR 8). The highest mean wind speed is achieved for WR 12, with 7.6±1.1 m.s**^−^** ^1^ and the lowest for WR 3 with 3.7±0.9 m.s**^−^** ^1^. The wind directions are mainly eastern, with the most southern one at 100.0±12.8° (WR 12) and the most northern one at 42.5±91.4° (WR 3) (Figure 4).

**Table 4:** Summary of the 12 wind regime characteristics.

ID cluster	Start date	Nb. of sequences per cluster	Wind regime probability (%)	Wind speed (m.s^−1^)		Wind direction (°)	
				Moy.	SD	Moy.	SD
1	28/08/81	135	11.2	7.1	0.9	96.1	11.3
2	16/03/82	98	8.1	4.6	1.9	87.6	66.2
3	20/12/84	87	7.2	3.7	0.9	42.5	91.4
4	14/05/86	83	6.9	4.9	1.8	94.3	51.1
5	24/05/86	94	7.8	5.9	1.5	93.2	18.8
6	12/01/94	83	6.9	4.2	1.4	75.8	63.7
7	22/01/94	88	7.3	4.8	1.4	81.3	61.6
8	02/05/94	160	13.3	4.4	1.3	80.9	37.7
9	06/01/99	108	9.0	5.7	1.2	89.0	17.6
10	07/03/99	86	7.1	6.2	1.2	94.6	20.0
11	06/04/99	122	10.1	5.4	1.3	87.8	14.1
12	25/09/08	59	4.9	7.6	1.1	100.0	12.8

### Analysis of the connectivity variance

The variance of the potential connectivity (*P_sd_*) (Table 5) is mainly related to the “Destination” (D) and “Spawning site” (S) factors that explain 59.4% and 25.9% of the total variance respectively. The interaction of (S : D) explains an additional 7.9% of the variance. Finally the PLD factor explains another 5% of the variance. Some other interactions have a significant effect on the connectivity variability. Namely (WR : D), (PLD : D), (WR : S : D) and (PLD : S : D), but with contributions all under 1%. The factors “Release level”, “Swim” and “Wind Regime” do not contribute significantly to the connectivity variance.

**Table 5:** Results of the variance analysis on potential and realistic connectivity.

		Potential connectivity			Realistic connectivity		
Factor	DF	*p* value	Signif.	*%Var*	*p* value	Signif.	*%Var*
Release Level (RL)	1	0.476		0.0	<2.10**^−^** ^16^	***	41.0
Swim (Sw)	1	0.883		0.0	0.935		0.0
Wind Regime (WR)	11	0.374		0.0	0.493		0.0
PLD	3	<2.10**^−^** ^16^	***	5.0	2.6.10**^−^** ^10^	***	1.3
Spawning Site (S)	11	<2.10**^−^** ^16^	***	25.9	<2.10**^−^** ^16^	***	2.9
Destination (D)	11	<2.10**^−^** ^16^	***	59.4	<2.10**^−^** ^16^	***	39.8
RL : S	11	0.389		0.0	<2.10**^−^** ^16^	***	8.4
PLD : S	33	6.13.10**^−^** ^6^		0.5	0.001	***	0.3
RL : D	11	0.706		0.0	4.10-4	***	0.4
WR : D	121	0.003	**	0.2	0.127		0.1
PLD : D	33	0.019	*	0.1	0.282		0.0
S : D	121	<2.10**^−^** ^16^	***	7.9	<2.10**^−^** ^16^	***	5.2
RL : S : D	121	0.871		0.0	0.013	*	0.2
WR : S : D	1331	0.040	*	0.1	0.430		0.0
PLD : S : D	363	2.94.10**^−^** ^8^	***	0.7	0.005	**	0.3
			*sum*	*99.96*		*sum*	*99.93*

Only significant interactions are listed. DF corresponds to the degree of freedom and *%Var* gives the percentage of the total variance explained by each factor.

Signif. codes : ‘***’ 0.001, ‘**’ 0.01, ‘*’ 0.05, ‘ ’ 1.

For the realistic connectivity (*L_sd_*), the factor “Release Level” (RL) alone explains 41% of *L_sd_* variance. More than the depth level of larvae release, this factor reflects the effect of broodstock density on connectivity, since the release level is not significant on the potential connectivity. The “Destination”, “Spawning Site” and “PLD” factors have a significant contribution to *L_sd_* variance, with 39.8%, 2.9% and 1.3%, respectively. The interactions (RL : S) and (S : D) contribute 8.4% and 5.2% of the variance, respectively. Finally, the interactions (PLD : S), (RL : D), (RL : S : D) and (PLD : S : D) contribute significantly to the realistic connectivity variance, but at less than 1%. The “Swim” and “Wind Regime” factors do not contribute significantly to the realistic connectivity variance.

### Spatial patterns of connectivity

**Figure 5:**
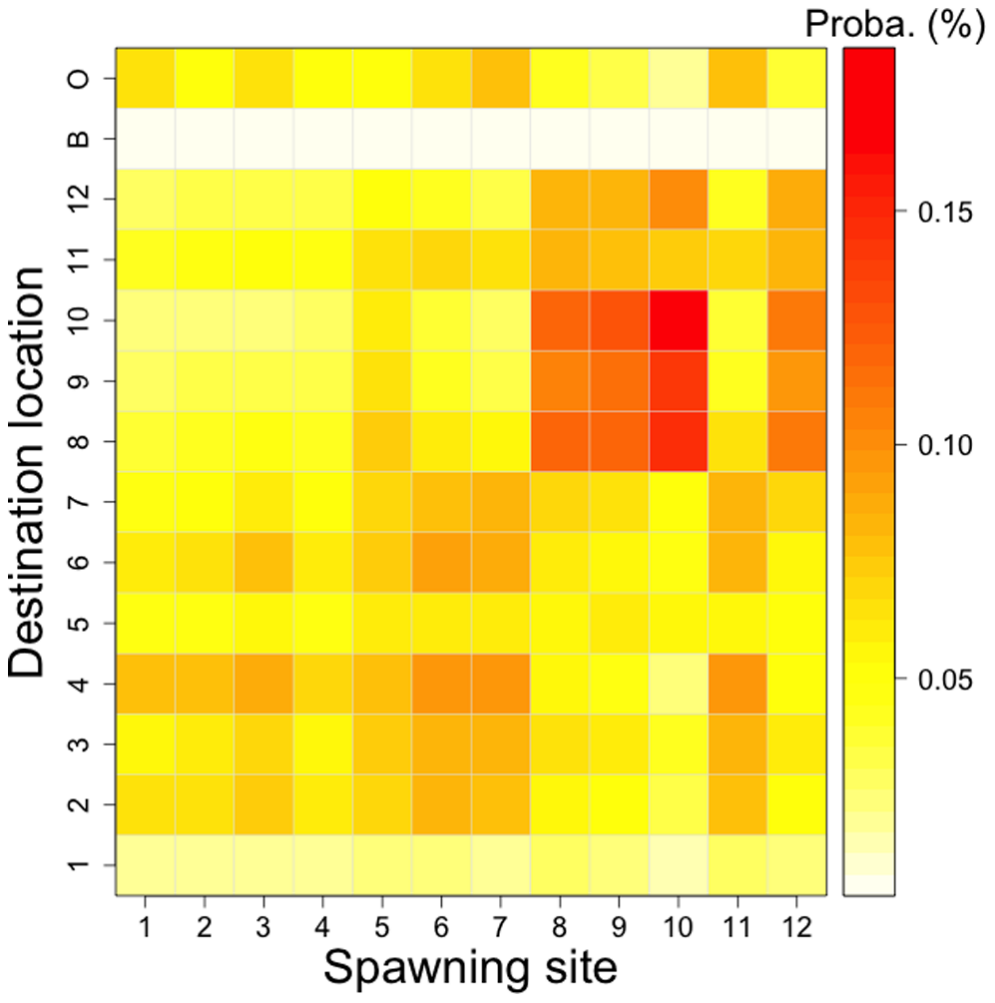
Potential connectivity matrix (*i.e.*, probability). This potential connectivity is an average of the 12 wind regimes scenarios. The potential connectivity is calculated as the ratio between the number of larvae in the destination location *j* after 20 days of dispersal, coming from the spawning site *i*, and the total number of larvae emitted in the spawning site *i*. In the destination locations, “B” represents the shallow waters and “O” the open ocean.

**Figure 6:**
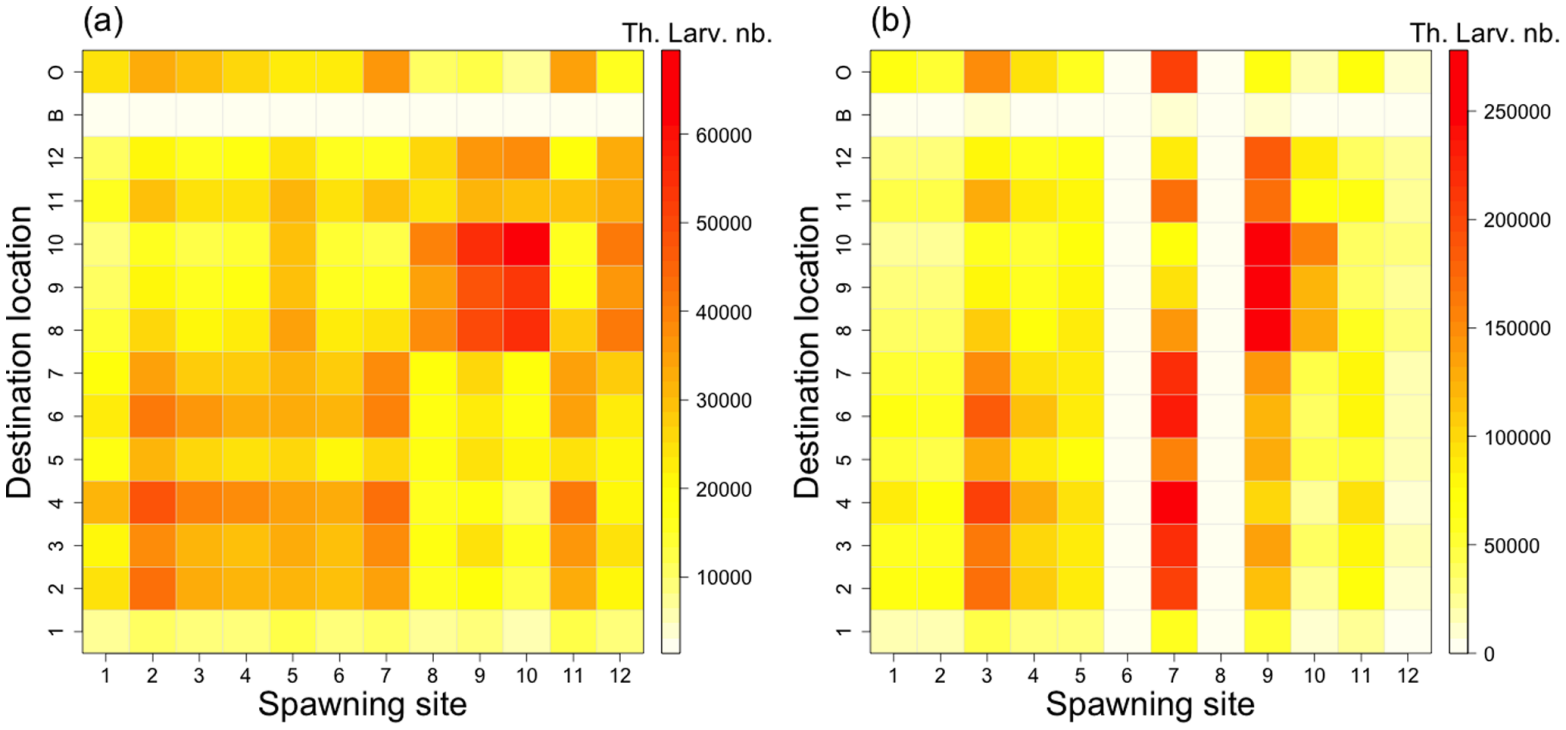
Realistic connectivity matrix (*i.e.*, theoretic larval number). The realistic connectivity corresponds to the mean potential connectivity (see Figure 5), spatially weighted in spawning sites by (a) the natural broodstock density and (b) the reared broodstock density, respectively. In the destination locations, “B” represents the shallow waters and “O” the open ocean.

The potential and realistic connectivity matrices computed after 20 days of simulation are presented in Figure 5 and Figure 6 respectively.

The intra-lagoonal potential connectivity (*P_sd_*) reaches 5.9±2.7% on average, giving a mean coefficient of variation (CV = SD/MV) of 45%. Patterns of *P_sd_* show a symmetry close to the diagonal, with two main sectors showing high retention: the northeastern part of the lagoon (i.e., sites 8, 9, 10 and 12), with highest values between sites 8, 9 and 10 (reaching 18%), and the southwestern part (i.e., sites 2, 3, 4, 6 and 7), with lower connectivity levels. The lowest connectivity is measured in the destination location (D) 1 and 5. The loss through export in the shallow waters appears very low, with 0.5% in average. However, the export through the pass to the open ocean averaged 5.2%, with higher level coming from spawning sites 7 and 11 (7.9% in average). Site 11 shows an asymmetric pattern since it exports a high number towards the southwestern destination locations and also receives larvae from the northeastern spawning sites.

The intra-lagoonal realistic connectivity (*L_sd_*) is respectively 25.9±11.3×10^3^ and 70.3±63.7×10^3^ in average for the natural and reared scenarios, giving a coefficient of variations of 44% and 91%, respectively. Patterns of realistic connectivity due to the wild oysters show an increase of the retention in the western sector mainly due to an increase in the contribution of spawning sites (S) 2 to 4. Conversely, connectivity level in the northeastern sector decreases. The realistic connectivity due to reared oysters shows a more asymmetric pattern. Spawning sites 6 and 8, without broodstocks, do not contribute to the global connectivity. Conversely, sites S 3, 7 and 9, with the highest broodstock densities, have their contributions reinforced in the reared scenario.

**Figure 7:**
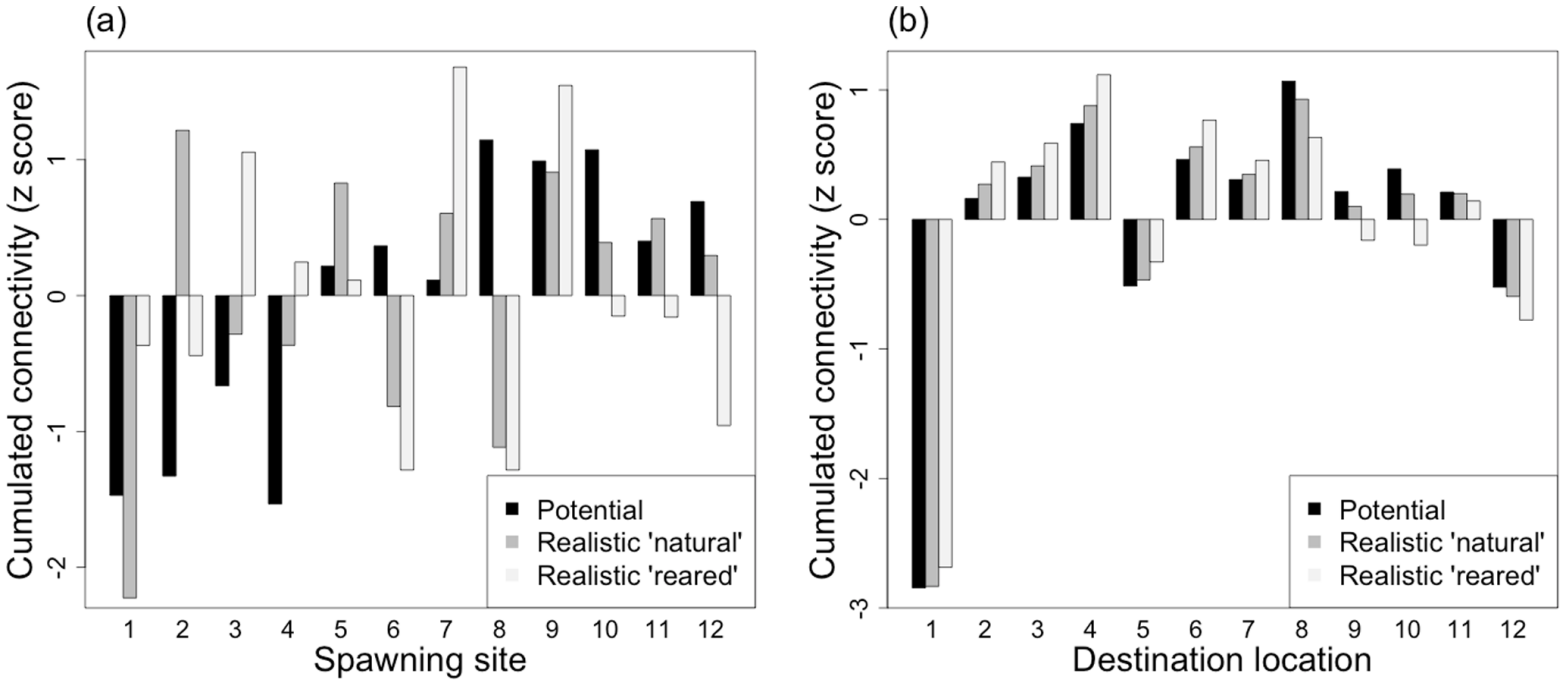
Cumulated connectivity according to (a) the spawning site and (b) the destination location. To compare results with different units, the cumulated connectivity for each scenario (potential and realistic: ‘natural’ and ‘reared’) is standardized using the standard score z.

The potential cumulated connectivity shows an increasing west to east gradient in spawning site contributions. Higher levels are recorded eastward, which is consistent with the higher water renewal rate of the western sectors (Figure 7a). However, this pattern is highly modified in the realistic cumulated connectivity, with an increase of the western sites contribution and a decrease of the eastern sites contribution for both the natural and reared scenarios. Only spawning site S1 shows a decreased contribution in the natural scenario. In the reared scenario, the three spawning sites previously identified (3, 7 and 9, with the highest broodstock densities), have an increased contribution while spawning sites S 6, 8 and 12 contribution is decreasing, due to the heterogeneity of their broodstocks.

The cumulated realistic connectivity in the destination locations (Figure 7b) suggests an increase of connectivity levels in the western destinations (i.e., from D 1 to D 7) and a decrease in the eastern destinations (from D 8 to D 12). These tendencies are strengthened in the reared scenario compared to the natural one. In potential connectivity, D 8 receives the highest number of larvae while this position is achieved by D 4 in the realistic scenarios, Conversely, D 1, 5 and 12 collect the lowest number of larvae in all scenarios.

### Influence of the PLD factor

**Figure 8:**
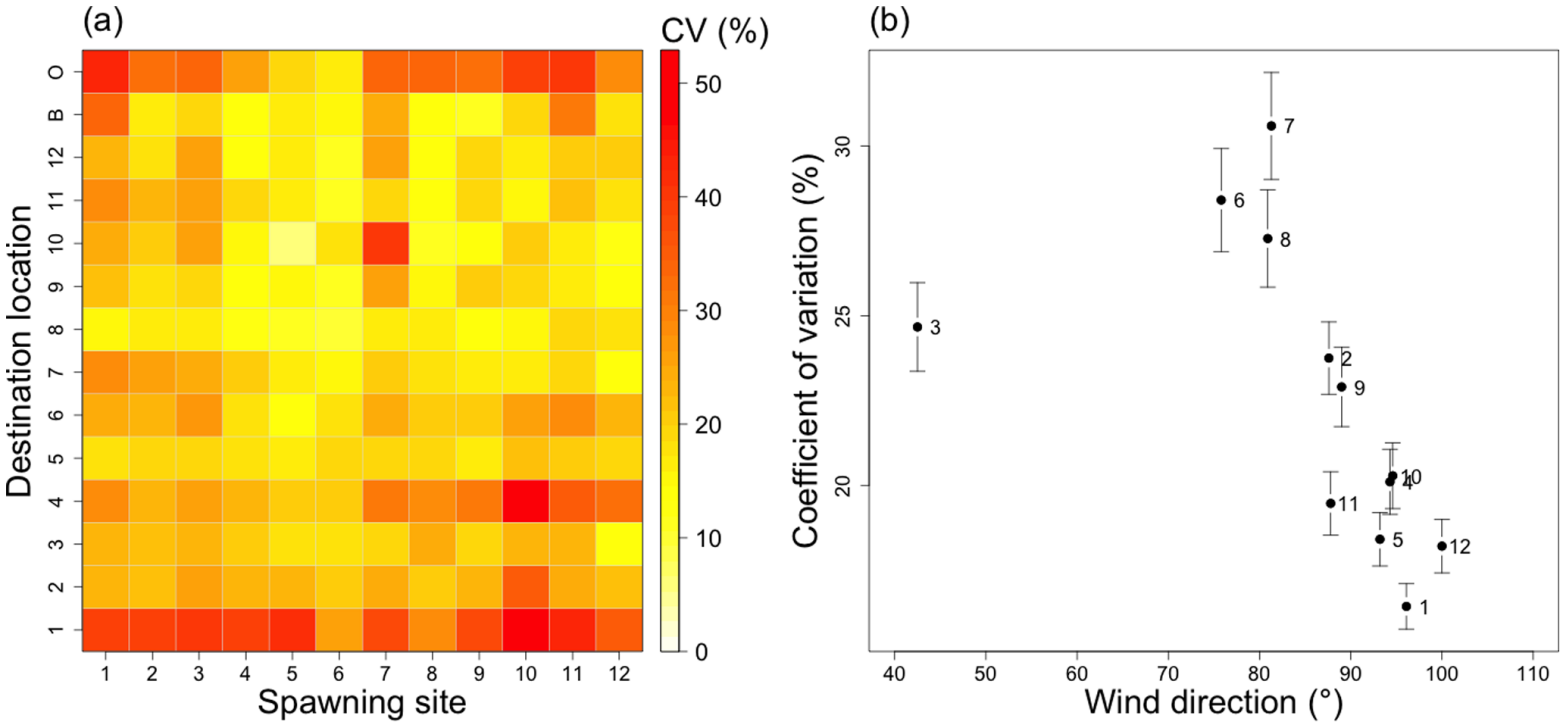
Connectivity variation (CV, %), according to PLD. The mean connectivity variations coefficients are calculated according to the PLD factor (*i.e.*, considering 15, 20, 25 and 30 days), by dividing the connectivity standard deviation calculated according to the PLD factor by the average. The matrix (a) is used to represent the spatial variability of CV, during mean wind conditions (average of the 12 wind scenarios). The graph (b) represents the CV variability according to wind direction. In the latter case, the error bars represent the spatial standard deviation. The numbers on graph (b) correspond to the wind regime indices (see Table 4).

The connectivity variation (CV) explained by the PLD factor is 23% on average (Figure 8). Figure 8 represents the spatial variability of this CV, in mean wind conditions, and the CV variability depending on the wind direction. Southwestern destination locations (DL 1 to 6) are thus the most variable (Figure 8a). In this sector, the DL 1 has the highest variation level according to the PLD, with a mean CV of 34%. The export rate toward the ocean is also highly dependent on the PLD factor, with in average CV = 31%.

Figure 8b provides the relation between the averaged wind direction of each wind regime (WR) and the mean coefficients of variation (CV) of the connectivity according to the PLD factor. A negative linear relation emerges following variations of wind directions from north to south. WR 3, the most northern wind, is an obvious outlier. The other northern winds have the highest connectivity variation, compared to east and southeast wind regimes.

### Seasonal patterns of connectivity

Despite little contribution of the wind regime (WR) to the connectivity variance, a significant effect of the interaction between the WR factor and destination location was measured (Table 5). Therefore, this interaction was further explored to identify a potential seasonal pattern.

**Figure 9:**
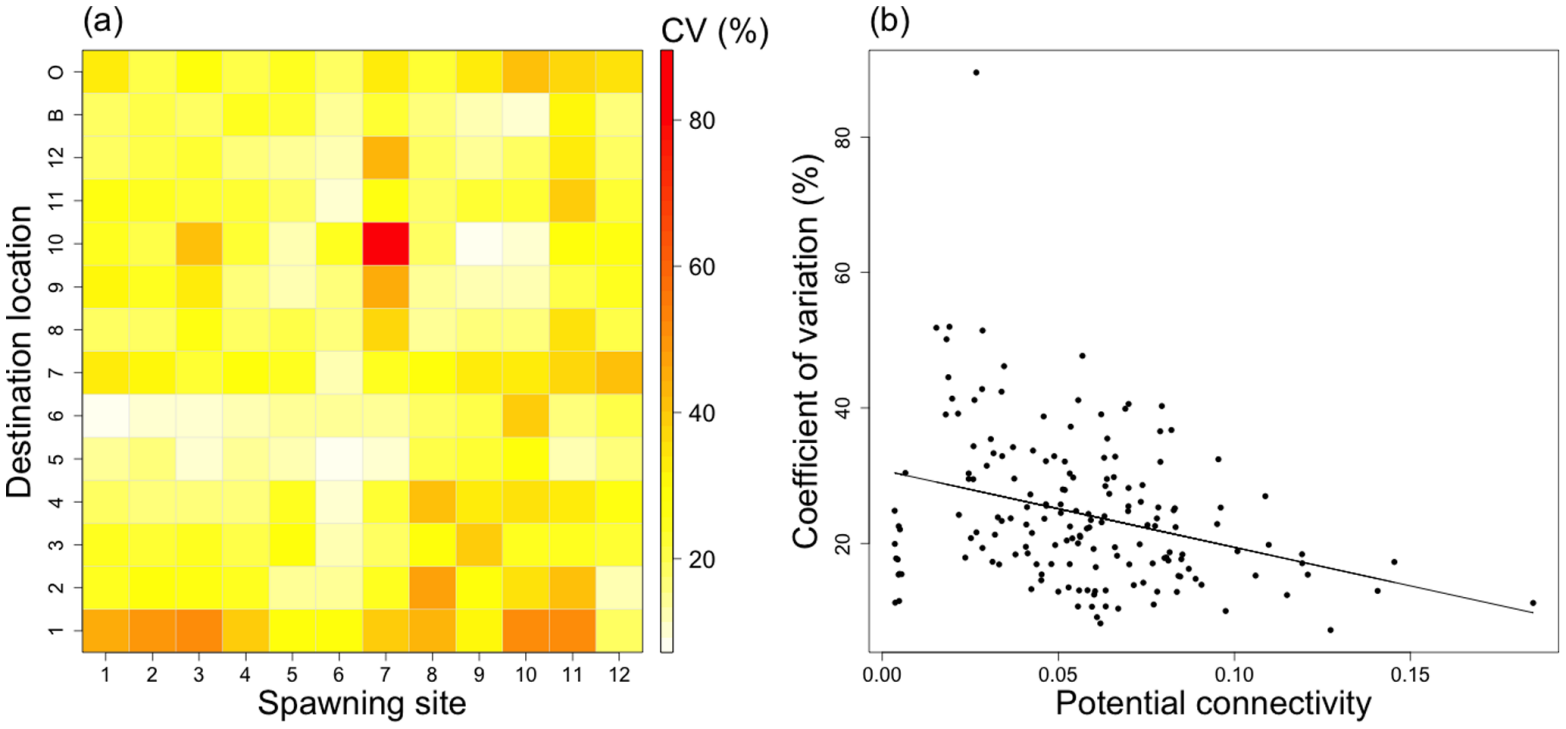
Connectivity variation (CV, %), according to wind. (a) Matrix of the connectivity variation and (b) relation between the potential connectivity and CVs. The coefficients of variation (CV, %) of connectivity were calculated after 20 days of simulations, by dividing the connectivity standard deviation calculated according to the wind factor (12 wind scenarios) by the average. In the destination locations, “B” represents the shallow waters and “O” the open ocean. In (b), the line is the linear regression.

Firstly, we investigated the spatial pattern of overall connectivity variation associated to the WR factor (Figure 9a). The overall average of CV is 24%. CV appears inversely related to the potential connectivity (Figure 9b). Eastern spawning sites, from S 7 to S 12, display the highest levels of variation, with an extreme for the S 7 and D 10 cell. This cell reaches 89% and is explained by a high level of connectivity only during northeastern winds (wind regime 3, data not shown). The destination location D 1 shows the highest level of variation (mean 40%). Conversely, D 5 and 6 have the lowest levels of variation with 16% and 17% on average, respectively.

**Figure 10:**
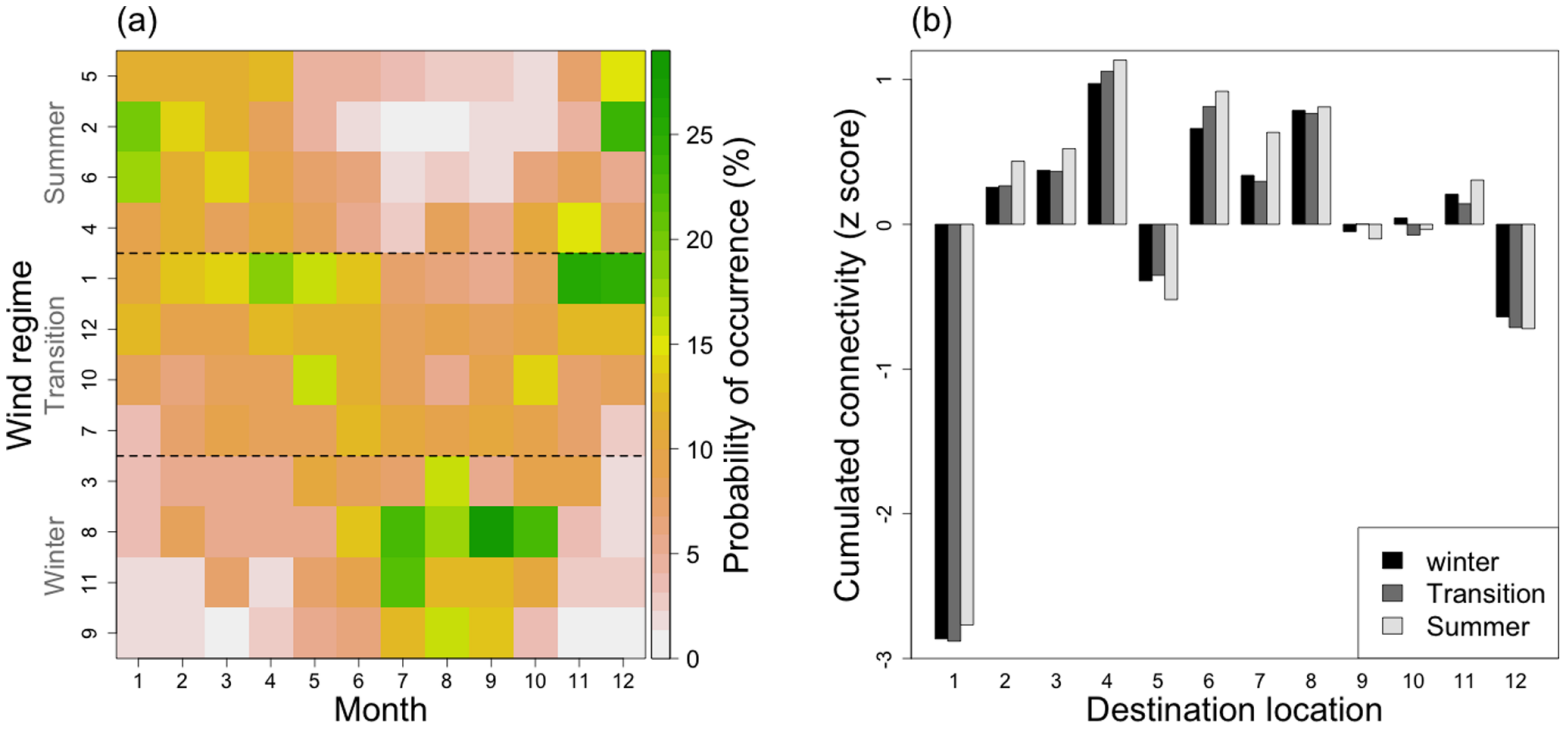
Seasonal pattern of connectivity. (a) Monthly probability of occurrence (in %) of each wind regime and (b) cumulated realistic connectivity, by season. The data used for realistic connectivity are the sum of the reared and natural scenarios. The connectivity is standardized using the standard score z.

Secondly, we explored the seasonality in connectivity patterns. The probability of occurrence of each wind regime in each month was calculated and the wind regimes were ranked to reveal their seasonal patterns (Figure 10a). Wind regimes: 3, 8, 9 and 11 are mostly observed in the austral winter (i.e., June to October) while regimes 2, 4, 5 and 6 are observed in the austral summer (i.e., November to May). Other wind regimes are present during transition periods, with regular occurrences all year long.

To evaluate how the destination locations may be favored during a specific season, realistic connectivity (*L_sd_*) was cumulated by destination location and averaged for each of the three periods identified (i.e., winter, transition, summer) (Figure 10b). The austral summer winds generate the highest connectivity levels, especially in the southwestern part of the lagoon (i.e., destination locations: 2, 3, 4, 6, 7 and 8). The austral winter and transition conditions create the lowest overall connectivity levels in the lagoon.

## Discussion

Understanding recruitment patterns across a broad range of scale is necessary to improve the management and conservation of marine communities [4], [36]. This is especially true for species used as a resource in a fishing or aquaculture context. Their population dynamics can lead to overfishing and quick decline in case of low recruitment. Very few studies have addressed recruitment patterns of mollusks in atoll lagoons [37] and this study gives new insights on the biological and physical factors affecting the dispersal and *in fine* the recruitment of one species: the pearl oyster *P. margaritifera*, which has a high commercial and social value in French Polynesia. In the present case, the entire pearl oyster production remains dependent on the natural collection of juveniles on artificial substrates [16]. In the last decade, spatial and temporal variations in spat collection, including poor production years, led to professional and institutional concerns and demand for knowledge and tools to improve practice [38]. After the first step of implementing, calibrating and validating with actual larvae counts a transport model [18], [19], this study refined the results by looking at how forcing variables act spatially and temporally on larval dispersal, and connectivity. These results have practical and management implications. In particular, the calculation of a realistic connectivity has shown the sensitivity of the larval supply to the location and density of the reared broodstock.

Not surprisingly, the spawning site, destination location and pelagic larval duration (PLD) are the main drivers of the connectivity patterns in Ahe atoll lagoon. In contrast, vertical swimming, depth of release and wind regime taken alone did not significantly contribute to the connectivity variability. Almost 60% of the potential connectivity variability was explained by the destination location factor only. This emphasizes the careful choice of collection areas for successful spat collection. Despite the simple saucer-shaped lagoon morphology, the larvae were far from being homogeneously distributed throughout the lagoon. In particular, the two northeastern and southwestern sectors had high retention levels and a low degree of connection between them. This is due to the two main barotropic circulation structures described by Dumas et al. (2012) [18]. The connection between the pass sector (i.e., site 4 in the west) and the eastern sector 11 (Figure 5) is itself explained by the downwind surface flow and upwind mid-depth return flow described in Dumas et al. (2012) [18].

The pelagic larval duration (PLD) of any species affects its broad-scale connectivity [39]. The PLD is also related to the growth rate of larvae, which itself depends on the environmental conditions (i.e., trophic state and temperature) [7]. In Ahe lagoon, because the currents are weak [18], larval dispersal is strongly dependent on the PLD even if the lagoon is small (i.e., 142 km^2^). Our results (Figure 8) show that PLD mostly modulates connectivity between the northeast and southwest parts of the lagoon. These connections are promoted by longer PLD and by northeastern winds. A linear relation between the wind orientation and the connectivity variation in time (Figure 8b) showed that the most southern wind quickly homogenizes larval concentrations, which rapidly leads to steady connectivity patterns. In contrast, a northern wind leads to slower homogenization and thus to more variable connectivity in time. Longer PLD also increase export rate, with thus a decrease in overall larval supply. Here, we considered PLD homogeneous in time and throughout the lagoon. However, *P. margaritifera* larvae growth rate can be spatially and temporally heterogeneous according to temperature, available trophic resources and their spatial distribution [7]. Depending on the dispersal pathways, the larvae may experience different trophic conditions leading to different growth patterns. This might reinforce the contribution of some of the spawning site locations and thus might change the connectivity patterns. Further work could seek to refine the dispersal model using a dynamic PLD in time and space.

Swimming behavior of larvae could also significantly control larval dispersal outcomes [6]. However, our results showed that the larvae dial vertical migration does not have any effect on the connectivity patterns. Here, larval behavior was not modulated spatially and temporally while several factors may play a role in the vertical migration of planktonic larvae (e.g., light, food, salinity discontinuity, temperature, predators, larval size/stage) [6], [40], [41]. We also did not change the behavior with larval size or development stage. Indeed, there is currently no evidence that *P. margaritifera* larvae change their behavior at each developmental stage. We are aware that *P. margaritifera* spat recruitment on collectors is at maximum 5 m depth and collapse deeper [17]. This may suggest a change of behavior at some late development stage but this remains poorly understood. A complementary sensitivity analysis performed on the maximum swimming speed (i.e., with a 3 times higher parameter α) did not provide significant effect (i.e., mean connectivity variation coefficient  = 0.2%, data not shown). This result reinforces our first observations and seems consistent with Kim et al. (2010) [41], who showed no significant effect of the swimming behavior on the *Crassostrea virginica* larvae dispersal in Mobile Bay, Alabama. These authors explained their results by frequent destratifications of the water column. In Ahe lagoon, vertical stratification, in temperature and salinity, is weak and transient [18]. The lagoon waters are well mixed [18] and homogenize larval concentrations in the water column. Vertical migration and turbulent mixing could also explain the similarity of connectivity patterns between bottom and surface releases, since larval concentrations can be quickly mixed.

This study suggests a small effect of the wind regime taken alone on connectivity patterns. This can be related to the relative constancy of the wind direction, coming mainly from the east combined with the simple geometric configuration of Ahe lagoon. Indeed, a consistent wind direction can not really generate complex patterns, in contrast to more complex embayment configurations like Poole Harbour where manila clam dispersal has been studied [42]. However, wind significantly contributes to the connectivity variability when coupled with destination location. The destination locations with the lowest connectivity levels are those showing higher variation coefficient when the wind regimes change. Transient unusual connections can occur, like, for example, between spawning site 7 and destination location 10 (i.e., south north connection). These locations are usually weakly connected but the infrequent northeastern winds strengthen the connections. Our study takes into account only the typical wind regimes occurring around Ahe. Regimes were defined statistically by clustering and from the barycenter of each cluster. Thus, we did not consider extreme events such as storms with wind coming from the west during 2 to 3 days [28]. Specific connectivity patterns related to extreme weather might exist, albeit with a low probability of occurrence during any given year.

The time scales over which larval connectivity varies is extremely important to understand the demographics of marine species [13]. In our study, the summer conditions gave the highest levels of larval supply in destination locations, mainly in the west of the Ahe lagoon. Winter and transition periods gave the lowest levels. The optimal dispersal period, giving the highest larval supplies is thus concomitant to the seasonal cycle of observed reproduction patterns. Indeed, reproduction occurs all year long, but with a maximum in austral summer [22], which is also the season with maximum observed larval concentrations and spat collection [17], [43]. However, inter-annual variability in the occurrence of each wind scenario, calculated over 32 years, indicates higher occurrences of winter winds with 9.9% in average, compared to summer and transition winds, with 7.4% and 7.6% in average, respectively (Table 4). These occurrences might be linked to large-scale climatological events, like the El Niño Southern Oscillation and La Niña conditions, which may affect physical processes (e.g., increased temperature, upwelling, circulation modification) and thus recruitment intensity [13], [44]. In the north Tuamotu sector, El Niño periods are associated with a lower wind directed further north, close to our summer conditions. Conversely, La Niña periods strengthen winds that shift more to the south, close to our winter conditions. El Niño conditions may thus give better dispersal conditions and increase the potential of recruitment throughout Ahe lagoon. However, other cross-effects (e.g., increased temperature) may increase predation or decrease food availability, thus negatively affecting the recruitment potential, and should have to be taken into account [44].

Our aim here was to gain a better understanding of the influence of the broodstock location on larval connectivity patterns. Taking into account the wild broodstock density and location as observed in Takapoto atoll by Zanini and Salvat (2000) [27], as well as the reared stock according to the rearing concessions, provided a more realistic view of the connectivity patterns. Even if the wild stock in Ahe was not exactly structured by depth range and as abundant as in Takapoto, the potential connectivity *vs* realistic connectivity results suggest that broodstock location play a major role in structuring the connectivity. More importantly, the reared broodstock contribution is three times higher than the natural one due to the high densities located on breeding concessions. The spatial distribution of reared stock, more heterogeneous, also induces a strong spatial variability of connectivity. However, both natural and reared scenarios resulted in an increase of the larval supply in western sectors. This result is consistent with professional practices that heavily rely on this sector for spat collecting.

Given the importance of the broodstock location on larval dispersal patterns, the influence of the age/size population structures between the different populations warrants further investigation. Indeed, farmers tend to favor young oysters to harvest pearls of superior quality [45]. This, in fact, promotes a cultured male stock since *P. margaritifera* is protandric hermaphrodite. This gives even more importance to the age and size distribution of the wild population. If the wild stock is declining, or has declined, a cultured stock massively dominated by male individuals may quickly lead to a collapse in spat collecting. Along with the sex/age population structure, the reproductive potential of the populations will also depend on food availability [46]. The introduction of mechanisms and factors that spatially explicit control reproductive effort is an interesting challenge for future modeling work [13]. For this reason, our next efforts will focus on the coupling of the current transport model with a bioenergetics growth and reproduction model.

According to Thomas et al. (2012) [19], the good agreement between larval concentrations simulations and observations suggests that most mortality occurs in the first 2 days of larval life. Hence, an estimation of this early mortality might allow a proper estimate of the real larval supply. The post-settlement mortality also needs to be better quantified. In their study, Friedman et al. (1998) [47] measured a spat mortality of 42% on collector, before harvest, mainly due to predation. This mortality has significant consequences on actual spat availability and also modifies the rearing practices (i.e., timing of harvest, rearing location, collector protection). These points, larval mortality and recruitment are also essential for future model parameterization and validation.

The various points discussed above call for additional research to fine tune the outputs according to a complex series of interacting factors. This will likely be a long path requiring additional field and experimental work. However, results already seem realistic and robust enough to think about generalizing the dedicated *Pinctada margaritifera* model to other important commercial and functional lagoon species. In principle, for other species with a larval dispersal phase, the same toolbox could be used, with adequate parameterization of PLD, stock location, swimming behavior sub-model and spatial units (source and sink sites). In particular, giant clams, found in abundance in several lagoons, are the focus of ongoing management models for fishery and aquaculture that could take advantage of lagoon-scale connectivity models [48], [49]. Beyond mollusks, lagoon-scale biophysical dispersal models could be used to assess the intra-lagoon connectivity of echinoderms such as sea cucumbers, the spread of invasive algae species in lagoons, the dispersal of pollutants and derelict aquaculture gear. A multi-species approach as described by Lòpez-Duarte et al. (2012) [50] may also lead to identify a range of biological traits (e.g., spawning time, PLD, behavior) and associated connectivity patterns for broad multi-objective management plans dedicated or not to bottom-dwelling cultured species in atolls and island lagoons. For passive drifters, such as invasive algae and pollutants, the Eulerian transport model and wind regime parameterization alone could help define accumulation risk zones very accurately. However, working in other atolls and lagoons than Ahe imply the acquisition of proper baseline data such as bathymetry, rim geomorphology, wave regime, and current measured in strategic places to set and validate the hydrodynamic model. Then other hydrobiological measurements may be needed according to the gradient of atoll morphology [51]. These are not trivial, simple and cheap tasks in logistically challenging remote places.

## Conclusion: Implications for Management

Understanding of the distribution of suitable spawning zones, larval transport processes and connectivity between spawning and nursery grounds are useful information for coastal spatial planning, management and fishery regulation. Our results are in agreement with professional farmers’ empirical knowledge, who, for instance, favor the western sectors as nursery grounds (Marine Resource Direction, pers. comm.). What the model brings that was not available before is a better understanding of why some sectors are suitable for spat collecting and others not, and why sectors that are efficient most of the time may be ineffective some years later. Farmers’ empirical knowledge after sometimes 30 years of practice is invaluable, yet, they still ignore the reasons leading to a good year and a bad year, and forecasting has proved to be unreliable from one year to another, with major economic consequences for some farmers. Here, the spatial variability of the connectivity (i.e., CV) was evaluated at 45% 44% and 91% for the potential and realistic (i.e., natural and reared scenarios), respectively. The current reared broodstock location appeared as the main source of variability in larval dispersal patterns, a fact that was not conceived by many who witnessed the start of pearl farming at time where the stock was only wild. The use of models is thus valuable as it brings critical new information to sustain the professional practices, improve their plans and collection strategies in the long run. In particular, broodstock location and the choice of collecting areas will determine the harvest performances and then the oyster supply for pearl production. As such, ongoing work includes the transfer of model outputs and possibility to query the model according to specific wind conditions to the French Polynesia Marine Resource Division.

The differences between the potential and realistic connectivity showed the significant contribution of the pearl oyster broodstock location to its own dynamics. As such, spawning sanctuaries are cited as an effective strategy to sustain oyster stock replenishment [52]. The selection of sanctuary areas, with an effective connectivity potential toward sectors traditionally used by professionals, and from a high reproductive potential broodstock (i.e., sex-ratio, maturation efficiency), may optimize spat collection. In Ahe atoll lagoon, the potential connectivity showed the high level of retention in two hydrodynamic cells. An increased broodstock in these two sectors should enhance spat collection potential. As fecundity is highly related to the trophic resource [46], sanctuaries should be located in the richest sectors. In Ahe, the highest levels of plankton concentration are found on the southwestern sector [53], and contribute to the larval supply in the collection area promoted by the farmers. The southwest area might be a suitable sanctuary, allowing an increase in the collecting yield by optimizing the population structure in this sector. In general, this work points to the need to rethink how the adult, non-reared stock, is managed. Instead of routinely discarding shells no longer used for pearl production, it may be wise to promote these sanctuaries in suitable areas. The idea is widely accepted as shown during the restitution of the first modeling work to farmers (in November 2010). The implementation is now in local managers’ hands.

Beyond Ahe, the present work offers modeling tools and connectivity concepts applied to a specific aquaculture context. Similar approaches could be applied to lagoons with different or more contrasted weather, different configurations, and higher or lower degrees of exchange with the ocean. The conceptual and technical bases can now be used for further applications in other lagoons, for other scientific and management issues.
